# Predictive Forwarding Rule Caching for Latency Reduction in Dynamic SDN

**DOI:** 10.3390/s25010155

**Published:** 2024-12-30

**Authors:** Doosik Um, Hyung-Seok Park, Hyunho Ryu, Kyung-Joon Park

**Affiliations:** 1 Department of Interdisciplinary Studies (Artificial Intelligence Major), Daegu Gyeongbuk Institute of Science and Technology (DGIST), Daegu 42988, Republic of Korea; uds0909@dgist.ac.kr; 2 Department of Electrical Engineering & Computer Science, Daegu Gyeongbuk Institute of Science and Technology (DGIST), Daegu 42988, Republic of Korea; hyungseok@dgist.ac.kr (H.-S.P.); ryuhyunho@dgist.ac.kr (H.R.)

**Keywords:** software-defined networking, mobile unmanned swarm node, predictive forwarding rule caching, dynamic network optimization

## Abstract

In mission-critical environments such as industrial and military settings, the use of unmanned vehicles is on the rise. These scenarios typically involve a ground control system (GCS) and nodes such as unmanned ground vehicles (UGVs) and unmanned aerial vehicles (UAVs). The GCS and nodes exchange different types of information, including control data that direct unmanned vehicle movements and sensor data that capture real-world environmental conditions. The GCS and nodes communicate wirelessly, leading to loss or delays in control and sensor data. Minimizing these issues is crucial to ensure nodes operate as intended over wireless links. In dynamic networks, distributed path calculation methods lead to increased network traffic, as each node independently exchanges control messages to discover new routes. This heightened traffic results in internal interference, causing communication delays and data loss. In contrast, software-defined networking (SDN) offers a centralized approach by calculating paths for all nodes from a single point, reducing network traffic. However, shifting from a distributed to a centralized approach with SDN does not inherently guarantee faster route creation. The speed of generating new routes remains independent of whether the approach is centralized, so SDN does not always lead to faster results. Therefore, a key challenge remains: determining how to create new routes as quickly as possible even within an SDN framework. This paper introduces a caching technique for forwarding rules based on predicted link states in SDN, which was named the CRIMSON (Cashing Routing Information in Mobile SDN Network) algorithm. The CRIMSON algorithm detects network link state changes caused by node mobility and caches new forwarding rules based on predicted topology changes. We validated that the CRIMSON algorithm consistently reduces end-to-end latency by an average of 88.96% and 59.49% compared to conventional reactive and proactive modes, respectively.

## 1. Introduction

In recent mission-critical environments, such as industry and military, unmanned vehicles are increasingly replacing human labor [1,2,3,4,5,6,7]. In industrial settings, unmanned aerial vehicles (UAVs), automated guided vehicles (AGVs), and autonomous mobile robots (AMRs) perform tasks such as material transport and sorting operations. Additionally, in the military field, exploration drones and military unmanned vehicles autonomously perform missions in dangerous conditions, such as battlefields or hazardous areas.

In this paper, we refer to unmanned vehicles that perform various missions as nodes. The system in which these nodes operate consists of a GCS (ground control system) and the nodes themselves [8]. The operation is maintained by various communications between the GCS and nodes [9,10]. The GCS sends data such as control commands and mission assignment information to the nodes. The nodes send collected data, such as sensor and location information, to the GCS. As various types of data are exchanged between the GCS and nodes, stable communication is necessary to ensure timely command delivery without delays and a communication environment that avoids data loss. Unstable communication causes data loss or increases network latency. This prevents the node from properly performing tasks or missions. In critical cases, the node encounters physical failures.

Using SDN in these conditions enables the creation of a low traffic communication system through centralized control [11,12,13,14,15]. Unlike the traditional communication methods, SDN is a method that separates and centralizes the control plane of each node. SDN allows traffic flow verification, processing, and monitoring through a central controller. Applying SDN in unmanned node environments enables efficient network traffic management. And by separating the control planes and reducing vendor dependency, it enhances integration among various types of nodes.

**Figure 1:**
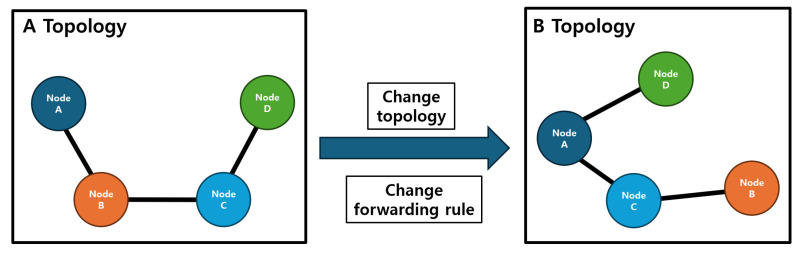
Necessity of forwarding rule updates in dynamic network. In a dynamic network, topology changes and link changes occur as nodes move around. Accordingly, forwarding rules for node-specific communication must be updated.

In SDN, when the topology changes, the SDN controller sends request messages, such as link layer discovery protocol (LLDP), to assess the network state. Therefore, in dynamic networks with node mobility shown in Figure 1, request traffic occurs frequently. A dynamic network indicates a state where the communication link status and topology change due to the mobility of the nodes [16,17,18]. In such an environment, SDN increases network latency due to the traffic generated by providing new forwarding rules. This leads to packet delivery delays, which can result in mission failure or physical node breakdowns. Therefore, it is necessary to make improvements to ensure low latency in dynamic networks.

Using caching can improve this issue. Caching refers to storing data in advance that is frequently used or expected to be used in the future [19,20,21,22]. By using caching, traffic and latency for data requests can be reduced. Therefore, in an SDN composed of mobile nodes, caching can help reduce the traffic and latency needed to create new forwarding rules. To achieve this, it is important to decide which information should be cached.

This paper proposes a caching technique for forwarding rules for predicted link states in SDN. The study aims to reduce latency and request traffic in dynamic networks compared to existing methods. For this purpose, we introduce the CRIMSON algorithm, which caches routing information in a mobile SDN optimized network. The CRIMSON algorithm is designed to detect changes in the state of network links based on node mobility, which is defined as a change in the communication link due to the movement of nodes in the network. These changes have the potential to affect the connectivity and topology of the network, thereby necessitating updates to forwarding rules in order to maintain stable communication. After a change is detected, it caches new forwarding rules based on the predicted topology changes. The CRIMSON process consists of three stages. The proposed algorithm is evaluated using a swarm node setting with UAV modeling, utilizing the open network operating system (ONOS) and Mininet-WiFi simulator. A swarm node is a network configuration consisting of N mobile nodes, typically UAVs, that operate in a decentralized manner, enabling cooperative communication and dynamic interactions for task execution. In the validation, CRIMSON’s latency and LLDP packet count are assessed. The results indicate that CRIMSON consistently maintains low latency under various bandwidth conditions. We validated that the CRIMSON algorithm consistently reduces end-to-end latency by an average of 88.96% and 59.49% compared to conventional reactive and proactive modes, respectively. Additionally, it was confirmed that CRIMSON has a lower LLDP packet count compared to the proactive mode. The results of this study confirm that CRIMSON can achieve low network latency and reduced traffic in SDN-based dynamic networks. The main contributions of this study are summarized as follows:

- This paper proposes the CRIMSON algorithm, which is a caching technique for forwarding rules for predicted link states in SDN. The study aims to reduce latency and request traffic in dynamic networks compared to existing methods.
- This study analyzes the mobility of the swarm with UAV modeling to predict the link state. For this purpose, movement is detected through node position data. When the swarm’s movement exceeds a specific threshold, the predicted link state is generated. Then, the forwarding rules reflecting the predicted link state are cached. This process consists of three steps in the CRIMSON algorithm.
- This study evaluates the performance of CRIMSON in dynamic networks through simulation. In this result, the CRIMSON algorithm consistently reduces end-to-end latency by an average of 88.96% and 59.49% compared to conventional reactive and proactive modes, respectively.

The structure of this paper is as follows. Section 2 reviews studies related to SDN, caching, and dynamic networks. Section 3 explains the background necessary for understanding this study. This section explains important ideas and content used in the study, such as SDN, methods for installing forwarding rules, and the confusion matrix. Section 4 describes the system model in which the proposed algorithm is applied. Section 5 explains the overall flow and each detailed step of the proposed algorithm, CRIMSON. Section 6 introduces the simulation process and parameter settings for comparing the performance of CRIMSON. It also explains the process and results of CRIMSON’s performance evaluation. Finally, Section 7 concludes the paper and discusses directions for future research.

## 2. Related Work

As network environments become more complex and varied, more research is focusing on improving network performance through SDN and caching. Additionally, studies are exploring network setups that use unmanned nodes.

As network settings become more complex, SDN research has been contributing to enhancing network performance and improving management efficiency in various industries. It has been validated in [23] that the centralized management of SDN reduces management complexity and improves network management efficiency in WSN (Wireless Sensor Network) contexts. To ensure reliability and flexibility between different types of wireless devices in IoT environments, ref. [24] applied SDN and a routing protocol using reinforcement learning, which reduced network latency and improved packet throughput. The benefits of applying SDN to WSN for network management, such as energy efficiency, dynamic network management, and routing optimization, are explained in [25]. It was shown in [26] that applying a centralized approach through SDN improved load balancing by up to 14% and enabled the processing of more network traffic. Applying SDN in military environments contributes to network status monitoring and enhanced security, as explained in [27]. Thus, using SDN provides advantages from a network perspective through traffic management and improved throughput via a centralized approach.

There is increasing interest in research aimed at achieving high communication performance in dynamic network settings. The software architecture and benefits of the prototype network called UAVNet, specialized for UAVs, are introduced in [28]. Sensor network auto-deployment and multi-hop communication are used to construct an IoT network in outdoor settings, as shown in [29]. The architecture and activation techniques for stable communication between aerospace and airborne systems, along with the optimization process for the performance analysis of dynamic networks, are described in [30]. A user-customized network that dynamically utilizes network resources by predicting voice call duration and data usage using LightGBM is introduced in [31]. Mobile network traffic prediction employs machine learning algorithms such as multi-layer perceptron (MLP), multi-layer perceptron with weight decay (MLPWD), and support vector machine (SVM). The performance analysis of the predicted values can be found in [32]. These methods are extensively utilized in machine learning for pattern prediction and network performance optimization. UAVs are used as relay nodes in a NOMA (Non-Orthogonal Multiple Access) network to enhance network performance, as demonstrated in [33]. The NOMA network is a multiple access technique that allows multiple users to share the same frequency channel by differentiating signals through power levels. As demonstrated in [34], the optimized placement of UAVs utilizing the DDPG (Deep Deterministic Policy Gradient) method, a reinforcement learning algorithm, has been shown to enhance communication performance by up to 86%. In dynamic networks, communication performance is improved through traffic prediction, node placement, or specific network strategies.

The importance of caching is increasing for enhancing communication performance and creating a stable communication. Cooperative caching based on mobility prediction (CCMP) was proposed in [20] to improve the performance of vehicular content-centric networks (VCCNs). This method predicts the movement area based on the past trajectory of mobile nodes to improve network performance. The importance of caching research in 5G wireless systems, cloud, and internet computing is emphasized in [21]. 5G mobile network performance was improved in [22] by caching content based on predicted user movement paths, reducing average latency and backhaul load. A learning algorithm designated PopCaching, which makes caching decisions based on content popularity, was proposed in [35]. Content popularity, which denotes the likelihood of specific data or content being frequently requested by network users, was effectively utilized by this algorithm, resulting in an increased cache hit rate and a performance improvement of over 40%, especially under limited cache capacity. A machine learning-based caching strategy called ELCache, which considers user mobility and interests to enhance QoE, was used in [36]. Therefore, by using caching to reduce average latency and traffic, network performance can be improved.

The integration of SDN with caching mechanisms has been demonstrated to be a powerful approach to improve network performance in dynamic and data-intensive environments [37]. SDN-based cooperative caching mechanisms reduce backhaul traffic, mitigate download latency, and improve data transfer rates in mobile multimedia networks [38]. These mechanisms leverage user mobility and probabilistic caching to optimize resource utilization and ensure seamless content delivery. This combination also provides significant benefits in IoT environments, such as reduced latency, increased energy efficiency, and better resource allocation. These results highlight the need for SDN-caching integration to ensure scalable, reliable, and efficient network management across a wide range of applications.

This paper proposes the CRIMSON algorithm, which is a caching technique to forward rules for predicted link states in SDN. The algorithm aims to reduce latency and request traffic in dynamic networks.

### Limitations of Previous Work

Previous studies utilizing machine learning and reinforcement learning for SDN optimization have demonstrated significant improvements, but they often face certain limitations:

- Scalability and Computational Overhead: Many reinforcement learning-based approaches, such as those described in [24], require substantial computational resources and extensive training data. This makes it challenging to apply them in real-time application in highly dynamic networks.
- Latency in Dynamic Environments: Machine learning-based methods, including [32], often involve exploration and learning phases, which can cause delays, especially in rapidly changing systems.
- Adaptability to node mobility: Approaches such as collaborative caching based on mobility prediction in [20] have been shown to effectively handle content delivery, but they lack adaptability in high mobility environments where link conditions change frequently.

CRIMSON addresses these limitations with a predictive caching mechanism tailored to dynamic SDN networks. Unlike existing approaches, CRIMSON leverages node mobility to pre-cache forwarding rules, significantly reducing latency and computational overhead. Furthermore, CRIMSON stands out by its focus on preemptive rule caching for topology changes, enabling more reliable communication in highly dynamic environments.

## 3. Background

In this section, we introduce the technologies and methods used for understanding this paper. In this context, we present SDN, dynamic networks, and forwarding rule methods. We also discuss concepts such as graph-based representations, shortest path algorithms, caching, and the confusion matrix.

### 3.1. Software-Defined Networking (SDN)

**Figure 2:**
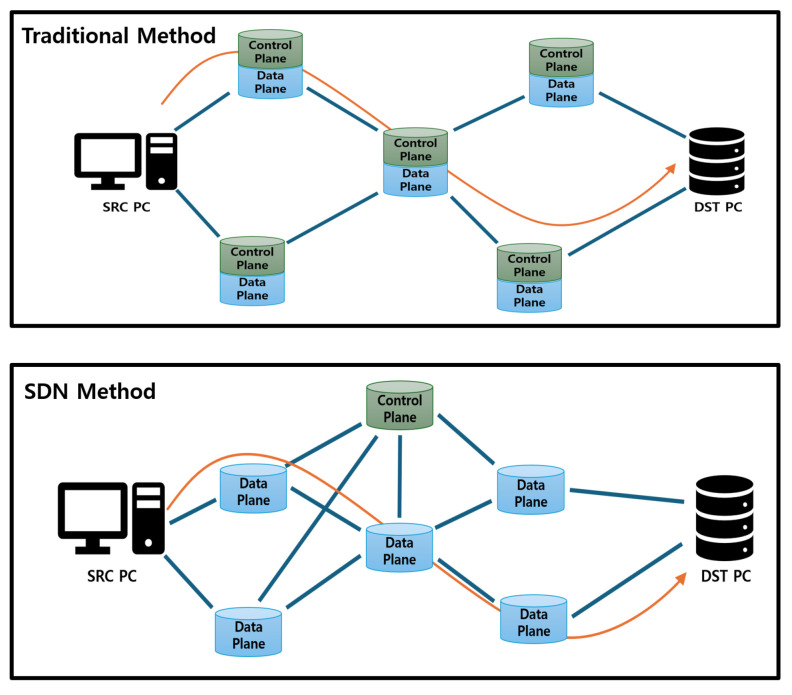
Comparison between traditional communication and SDN methods. In a traditional network, a control plane is configured on each node. However, in an SDN environment, only the central controller has a control plane. In this case, the central controller provides the forwarding rules.

SDN is a network management technology that divides the control and data-forwarding functions, enabling centralized control [11,12,13,14]. Unlike legacy network architectures, SDN allows applications for network management to be developed through the SDN controller, as shown in Figure 2. This enables the simple and flexible management and optimization of network resources. The components of SDN can be divided into the control plane and the data plane. The control plane handles tasks such as setting communication paths and managing topology. The data plane is the part that sends data according to the paths set by the control plane.

There are various SDN controllers, each offering unique features and functions [39,40,41]. The main types of SDN controllers are open network operating system (ONOS), OpenDaylight, Ryu, Floodlight, and Pox. Of these, the ONOS controller helps manage data flow and has the ability to scale for large networks. These features improve traffic management and provide benefits for cooperation in the industry through open-source communities.

**Figure 3:**
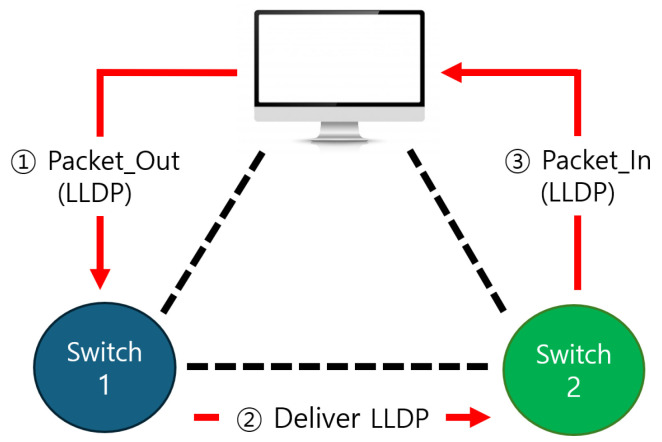
LLDP transmission process for communication between SDN nodes. In an SDN, the SDN controller recognizes new switches through the process of packet-out, deliver LLDP, and packet-in to the switches it already knows.

In SDN, the link layer discovery protocol (LLDP) is a protocol that allows network devices to recognize each other and check their connection status [42,43]. LLDP regularly shares information between devices in an SDN system to collect and maintain the network topology. The basic SDN flow includes incoming packets and checking the flow table. When a packet arrives at a network switch, the switch checks the flow table to determine how to handle the packet. If the flow table contains a forwarding rule for the packet, the switch processes the packet according to the stored rule. However, if there is no forwarding rule, the switch requests the appropriate forwarding rule from the SDN controller. SDN uses LLDP to explore the network topology and detect state changes, as shown in Figure 3.

### 3.2. Dynamic Network

A dynamic network is a network environment in which the topology continuously changes because of changes in node positions and link states [16,17]. Unlike static networks, dynamic networks require real-time management and adaptation because of the rapidly changing network conditions. One example of a dynamic network is mobile cluster node settings, where nodes move and communicate with each other. The dynamic network has issues such as link disconnection, network overhead, latency reduction, and path reconfiguration due to changing topology. This requires efficient management and optimized communication techniques because it causes high uncertainty, such as fluctuating link states and unpredictable traffic changes, leading to performance issues. A dynamic network changes in real time, so adaptive routing algorithms are essential.

### 3.3. Forwarding Rule Installation Method

The method of establishing forwarding rules has a significant impact on data transmission and the efficient use of network resources. This method is classified into reactive mode and proactive mode [44,45].

#### 3.3.1. Reactive Mode

Reactive mode is a method where a path is created in real time to handle a packet when it arrives. This approach finds and sets up a path only when specific data need to be sent. The advantage of this method is that it saves network resources because it does not need any paths set up in advance. But it takes a long time to send the first request and obtain a response. In a dynamic network, nodes are constantly moving, which requires replacing forwarding rules after they are requested. This lead to more network overhead and potentially increases network latency.

#### 3.3.2. Proactive Mode

The proactive mode is a method where all nodes in the network keep and manage routing information in advance. The proactive mode keeps updating the communication paths in the network. It also sets up forwarding rules between the sender and receiver before any data are sent. This method has the disadvantage of using computing power and network resources to keep and update routing information. However, it enables fast data transmission through pre-configured paths, ensuring stable communication, such as in environments where there is minimal communication delay and low data loss.

### 3.4. Graph Form Representation

In a network, the graph data represent node information as vertices and the connections between nodes as edges in the network information. This method is useful for representing network topology and analyzing the relationships between nodes. Graphs are represented in two ways, which are the adjacency matrix and the adjacency list [46].

**Figure 4:**
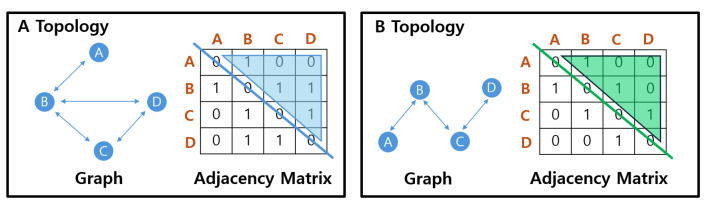
Representation of node link states using adjacency matrix. Graph data for each topology are represented as an adjacency matrix. Depending on the number of nodes (*n*), an n×n matrix is formed, where each row and column data represent the connection status between nodes.

An adjacency matrix represents the connection status between vertices of a graph in a two-dimensional array format. This array is an n×n matrix, where *n* represents the number of nodes (vertices) in the graph. Each element in the matrix represents whether there is a direct connection (edge) between two vertices. As shown in Figure 4, A[*i*][*j*] = 1 means there is an edge from vertex *i* to vertex *j*, whereas A[*i*][*j*] = 0 indicates that no edge exists between these vertices. On the other hand, an adjacency list stores a list of other vertices connected to each vertex. For each vertex, the list records the direct connections between the vertices. When comparing adjacency matrices and adjacency lists, adjacency matrices enable a quick validation of edge existence due to their lower time complexity. In particular, checking whether an edge exists between two nodes in an adjacency matrix can be performed in constant time O(1), since the presence or absence of a connection is stored directly in the matrix. It also offers advantages to understanding the graph structure.

### 3.5. Shortest Path Algorithm

The shortest path algorithm assists with finding the shortest way between two points, and it is one of the main topics in graph theory [47,48]. These algorithms are widely used in various fields, such as network routing, path finding in maps, and communication network design. There are different kinds of shortest path algorithms, and each one has its own features and purposes. The most well-known ones are Dijkstra’s algorithm and the Bellman–Ford algorithm.

While the A*’s algorithm has the advantage of reducing unnecessary node expansions, it has been shown to have performance issues in complex environments [49]. Dijkstra’s algorithm is a useful method for quickly finding the shortest path from one starting point with non-negative edge weights. The algorithm starts by setting the distance from the source vertex to zero. Next, it includes the steps of initialization, vertex selection, and distance updating. The initialization process means storing unprocessed vertices in a priority queue. Vertex selection refers to choosing the vertex with the shortest distance among the unprocessed vertices. Distance updating means calculating and updating the distances to all adjacent vertices connected to the selected vertex. This process repeats until the shortest path is determined. The bidirectional Dijkstra’s algorithm explores in two directions simultaneously and therefore performs better in terms of time and path-finding efficiency than unidirectional exploration with fewer node expansions. Unlike Dijkstra’s algorithm, the Bellman–Ford algorithm can be applied to graphs with negative edge weights. This algorithm works by cycling through each edge and updating the path if a shorter path is found. Compared to other shortest path algorithms, Dijkstra’s algorithm offers advantages in efficiency and simplicity for solving single source problems in graphs with non-negative weights.

### 3.6. Caching

Caching is a method used in computers and network systems to temporarily save data, so it enables quick access to necessary data when needed [19,20,21,22]. This method reduces data access time and improves overall system performance. As a result, it becomes a widely used optimization technique in various fields. In the network field, caching enables the reduction of network load by saving traffic and bandwidth, so it also leads to lower operating costs.

To make caching effective, it must have a high hit rate, which means the predicted data are very similar to the actual data. So, it is important that cached data keep a high hit rate, and this requires intelligent caching strategies. These approaches include methods for caching frequently used data as a priority or methods for caching predicted future data. These strategies enhance the efficiency of caching and optimize the use of system resources. This increases the reliability of caching and ensures the accuracy of data in the system.

### 3.7. Confusion Matrix

A confusion matrix is a matrix used to compare predicted values with actual values to measure the performance of a prediction model [50,51]. This matrix is widely used to evaluate how effectively a classification model works, especially in binary classification tasks. In the matrix, T represents True, F represents False, P represents Positive, and N represents Negative. So, TP means True Positive, TN means True Negative, FP means False Positive, and FN means False Negative.

**Figure 5:**
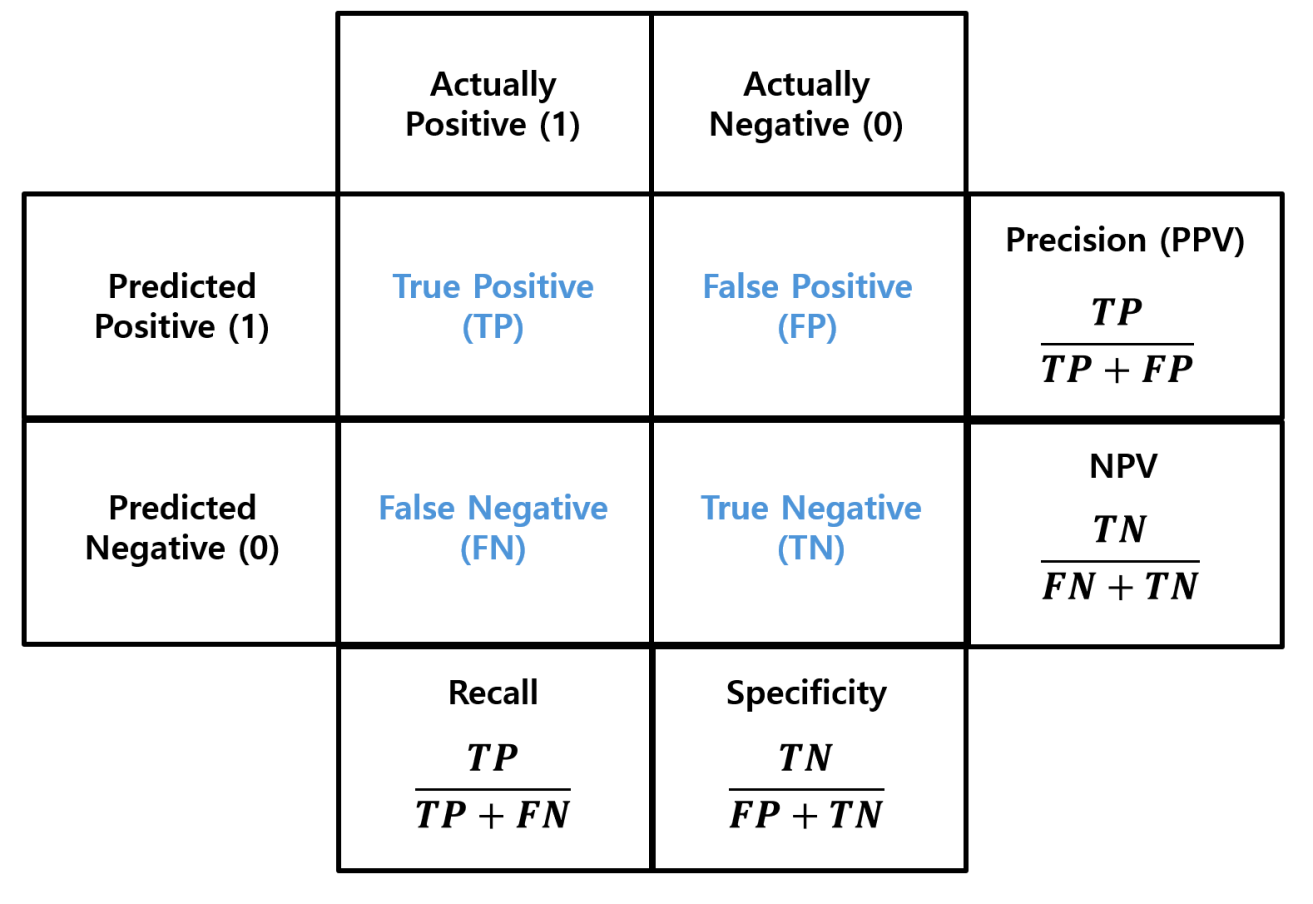
Confusion matrix. The confusion matrix calculates Precision, NPV, Specificity, and Recall using the TP, TN, FP, and FN metrics. This matrix is used to evaluate the performance of classification models.

As shown in Figure 5, various performance metrics are calculated from this confusion matrix. These include four key indicators: Precision (Positive Predictive Value), Recall, NPV (Negative Predictive Value), and Specificity. These metrics are defined as the ratios of the specific components in the matrix as follows:

$$\mathrm{Precision}=\frac{\mathrm{TP}}{\mathrm{TP}+\mathrm{FP}},$$

$$\mathrm{Recall}=\frac{\mathrm{TP}}{\mathrm{TP}+\mathrm{FN}},$$

$$\mathrm{NPV}=\frac{\mathrm{TN}}{\mathrm{TN}+\mathrm{FN}},$$

$$\mathrm{Specificity}=\frac{\mathrm{TN}}{\mathrm{TN}+\mathrm{FP}},$$

where TP is True Positive, TN is True Negative, FP is False Positive, and FN is False Negative.

The meanings of each metric are as follows. Precision means the ratio of correct predictions in all cases where the model predicted positive. Recall means the ratio of actual positives that were correctly predicted by the model. NPV means the ratio of correct predictions in all cases where the model predicted negative. Specificity means the ratio of actual negatives that were correctly predicted as negative by the model. These metrics evaluate different aspects of prediction performance. And based on the situation, a specific metric may be prioritized over others.

## 4. System Model

**Figure 6:**
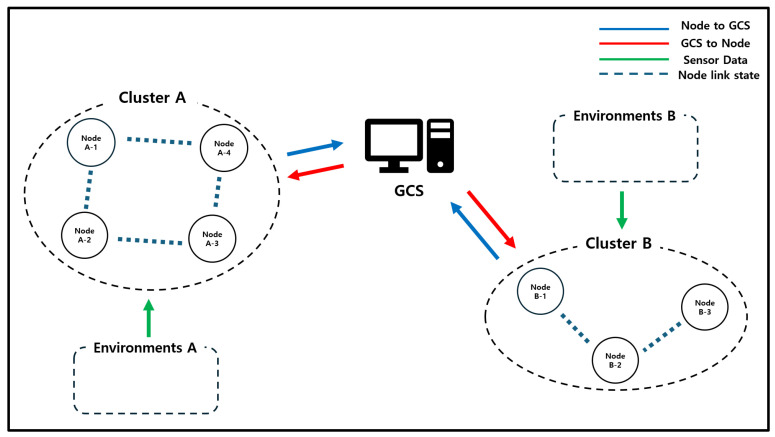
System model. This environment includes a GCS and multiple mobile unmanned nodes. The GCS and nodes transmit various types of communication, such as data collection, topology maintenance, and command control.

The CRIMSON algorithm operates in the environment shown in Figure 6. This setting consists of a GCS and a total of N mobile nodes. The GCS functions as an SDN controller and provides each node with forwarding rules. The N nodes change network link states according to their missions and create a dynamic network. In this environment, the nodes receive the necessary forwarding rules for communication from the GCS and perform network communications. These nodes include various types of devices, such as UAVs and UGVs, depending on specific missions or roles. This setting has different types of nodes that work together to complete missions. Additionally, due to mission situations and mobility, there is a possibility that all nodes may enter a multi-hop state where they are not directly connected to the GCS.

In this context, important information is exchanged in both directions between the GCS and the nodes [1,9,10]. The data flow in this system is divided into three main parts. The first type of data is sent from the GCS to the nodes, including control commands and mission assignment information. This type of data has a direct impact on mission performance and is time-sensitive. The second type of data is sent from the nodes to the GCS, including position information and sensor data. These data are used by the GCS to monitor the mission status in real time and make decisions. The third type of data is shared among the nodes, including situational analysis and risk information. These data help nodes understand the environment and respond collaboratively.

In this environment, a large amount of data is continuously transmitted between the GCS and the nodes and among the nodes. Therefore, environments with communication delays or a high risk of data loss are likely to impact mission performance. Delays or the loss of important control commands and situational information affect the system’s response and reliability. Consequently, if nodes fail to respond quickly to situational changes, it can result in mission failure or physical damage to the nodes.

The CRIMSON algorithm maintains low network latency and low request traffic in SDN-based dynamic networks. The CRIMSON algorithm caches forwarding rules based on predicted link states that reflect node mobility. By storing changes in advance before communication between nodes is interrupted, it keeps communication stable.

## 5. The CRIMSON Algorithm

**Figure 7:**
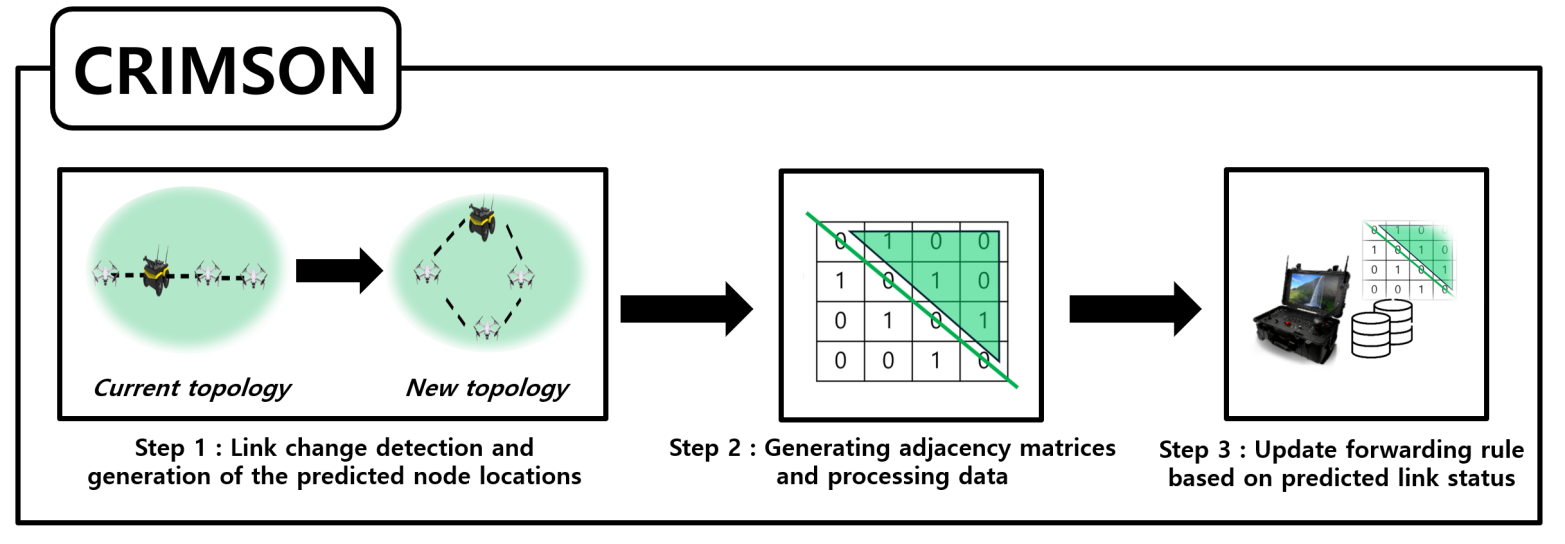
CRIMSON flow. CRIMSON is composed of three main steps. The first is topology change detection and generation of the predicted node locations. The second is the creation of a predictive adjacency matrix. The third is caching the forwarding rules for the predicted link states. Through this process, CRIMSON prepares forwarding rules in advance, reflecting the predicted link states.

**Figure 8:**
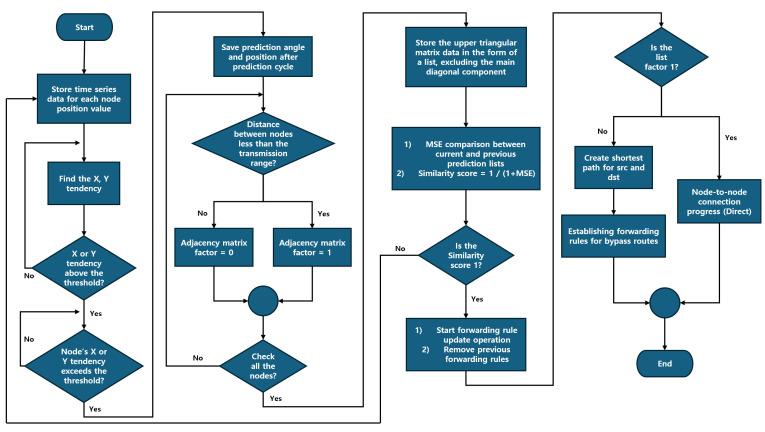
CRIMSON flow chart. The analysis of time-series data calculates node movement trends, and if they exceed a threshold, the system predicts the node positions. The system calculates distances between nodes using the predicted positions and checks them against the communication range to generate an adjacency matrix. The matrix updates forwarding rules for both direct and alternative paths.

In this section, we propose CRIMSON, a caching technique for forwarding rules for predicted link states in SDN. The overall scenarios for CRIMSON are shown in Figure 7, and the flow chart is shown in Figure 8. The simulation process generally consists of two parts. First, topology changes occur due to the mobility of nodes. For example, a change occurs from the current topology (e.g., Formation A) to a new unfixed topology (e.g., Formation B). Second, the proposed CRIMSON algorithm operates. The CRIMSON algorithm includes processes such as link change detection and node prediction location calculation, generating adjacency matrices and processing data, and updating the forwarding rule based on the predicted link status. The proposed method analyzes various scenarios that occur in dynamic networks through the simulation setup. This approach aims to quickly respond to changes in communication link status and topology. This reduces communication delays in dynamic networks.

### 5.1. Link Change Detection and Node Prediction Location Calculation

The description of Algorithm 1 is as follows. In this process, we calculate the predicted positions of nodes within the cluster in the X-axis and Y-axis directions. Then, we detect the mobility of the cluster. Through this, we predict changes in network link status and cluster position.

To achieve this, we first store time-series data for each node’s position in the cluster at each prediction cycle. These data enable us to monitor how the positions of nodes in the cluster change over time. Next, we compare the changes in the X and Y directions for each node in the cluster relative to their previous positions. This change from previous positions is calculated by the difference in the X and Y coordinates between prediction cycles. After that, we aggregate this information to identify a common movement trend for the cluster. Through this process, we calculate the average distance the nodes in the cluster move, allowing us to understand the movement trend of the cluster.

These movement trend data provide a reference point to identify the overall movement direction and rate of change for the cluster. We check whether the movement trend data of the cluster exceed a certain threshold. If the X-axis or Y-axis value of the movement trend exceeds the threshold, the cluster is predicted to be moving.

If it is predicted that the cluster will move, we perform a process to predict the positions of the nodes in the cluster after the prediction cycle. The calculations for each node’s predicted position are based on the node’s previous and current data. This includes calculating the angle by considering the movement direction and quadrant direction relative to the previous location. Then, we combine each node’s angle data and speed data to determine the expected position for each node. This process is repeated until the cluster’s movement tendency decreases below the threshold. This approach allows us to ensure the flexibility to quickly respond to changing environments by detecting cluster mobility in advance.
**Algorithm 1** Predict topology change and calculate node expected location  1:Save time series data for position values for each AP  2:Calculate X and Y tendency for clustered nodes**Calculate X Tendency:**  3:$x\_tendency=\frac{1}{\mathrm{len}(\mathrm{Number}\;\mathrm{of}\;\mathrm{Nodes})}\sum_{i=1}^{Number\;of\;Nodes}\left|\frac{\mathrm{Tendency}\_\mathrm{to}\_\mathrm{move}\_\mathrm{ap}[i]}{\mathrm{EX}\_\mathrm{Tendency}\_\mathrm{to}\_\mathrm{move}\_\mathrm{ap}[i]}\right|$**Calculate Y Tendency:**  4:$y\_tendency=\frac{1}{\mathrm{len}(\mathrm{Number}\;\mathrm{of}\;\mathrm{Nodes})}\sum_{i=1}^{Number\;of\;Nodes}\left|\frac{\mathrm{Tendency}\_\mathrm{to}\_\mathrm{move}\_\mathrm{ap}[i]}{\mathrm{EX}\_\mathrm{Tendency}\_\mathrm{to}\_\mathrm{move}\_\mathrm{ap}[i]}\right|$  5:**if** x_tendency>threshold **or** y_tendency>threshold **then**  6:    **for** each AP *i* **do**  7:        Determine quadrant direction  8:        Calculate prediction position using angle calculation  9:        **if** $\mathrm{AP}{\left[i\right]}_{x\_position}>\mathrm{threshold}$ **or** $\mathrm{AP}{\left[i\right]}_{y\_position}>\mathrm{threshold}$ **then**10:           $\mathrm{Save}\_\mathrm{Node}\_\mathrm{Angle}\left[i\right]=\tan\left(\frac{\mathrm{Tendency}\_Y}{\mathrm{Tendency}\_X}\right)$11:           $\mathrm{Predict}\_x\_\mathrm{position}\left[i\right]=x\_position+\mathrm{predict}\_\mathrm{move}\_\mathrm{distance}\cdot \cos\left(\pi \cdot \left(\frac{\mathrm{degrees}(\mathrm{Save}\_\mathrm{Node}\_\mathrm{Angle}[i])}{180}\right)\right)$12:           $\mathrm{Predict}\_y\_\mathrm{position}\left[i\right]=y\_position+\mathrm{predict}\_\mathrm{move}\_\mathrm{distance}\cdot \sin\left(\pi \cdot \left(\frac{\mathrm{degrees}(\mathrm{Save}\_\mathrm{Node}\_\mathrm{Angle}[i])}{180}\right)\right)$13:        **end if**14:    **end for**15:**end if**

### 5.2. Generating Adjacency Matrices and Processing Data

The description of Algorithm 2 is as follows. In this process, we determine the expected communication link status and topology after the prediction cycle. Then, we continue with saving graph data that reflect the predicted link status. To accomplish this, we first use the predicted positions of the nodes in the cluster after the prediction cycle.

Each node compares its own communication range at the predicted position with that of other nodes, excluding itself. Then, each node calculates which other nodes it will connect to based on the communication link status. If the distance between nodes is less than the specified communication range, it is predicted that a communication link will be created between the nodes. But if the distance is greater than the specified communication range, it is predicted that there will be no communication link between the nodes. This predicted communication link status for each node is represented by 0 or 1, where 0 indicates no communication link, and 1 indicates a communication link. This process is performed for each node from (*i* + 1) to n times, assuming the current node is the *i*-th out of n nodes.

The predicted link status for each node is represented as an adjacency matrix, which is created through this process. This matrix is represented as an n×n matrix if the number of nodes is *n*. To make calculations easier, we save these data as a list in the form of an upper triangular matrix, excluding the main diagonal elements.

Then, we compare this list of data to the previously calculated predicted link data using the mean squared error (MSE) method. This comparison enables us to find any differences between the two datasets. The similarity between the datasets is expressed as a similarity score, which enables us to determine if there has been a change in the network state. If there is a difference in the similarity score, we expect the network link status to change.
**Algorithm 2** Implementing expected adjacency matrix and processing data  1:Initiate the process of generating adjacency matrices for predicted link status  2:**for** each AP *i* **do**  3:    **for** each AP *j* where j>i **do**  4:        Calculate distance between AP*_i_* and AP*_j_*  5:        $d_{ij}=\sqrt{{\left(\mathrm{Difference}\;\mathrm{in}\;X\;\mathrm{of}\;\mathrm{AP}i,j\right)}^{2}+{\left(\mathrm{Difference}\;\mathrm{in}\;Y\;\mathrm{of}\;\mathrm{AP}i,j\right)}^{2}}$  6:        **if** $d_{ij}\leq \mathrm{Transmission}\;\mathrm{Range}$ **then**  7:           A[i][j]=1  8:           A[j][i]=1  9:        **else**10:           A[i][j]=011:           A[j][i]=012:        **end if**13:    **end for**14:**end for**15:Extract upper triangular part of adjacency matrix excluding the main diagonal16:Simple_Predict_arr=[]17:**for** each *i* from 1 to *n* **do**18:    **for** each *j* from i+1 to *n* **do**19:        Simple_Predict_arr.append(A[i][j])20:    **end for**21:**end for**22:Calculate mean squared error (MSE) between current and previous link prediction data23:$mse=\mathrm{mean}({\left(\mathrm{Simple}\_\mathrm{Predict}\_\mathrm{arr}-\mathrm{EX}\_\mathrm{Simple}\_\mathrm{Predict}\_\mathrm{arr}\right)}^{2})$24:Ex_Simple_Predict_arr=Simple_Predict_arr25:Calculate similarity score26:$similarity\_score=\frac{1}{1+mse}$27:**if** similarity_score≠1 **then**28:    Detect link changes and update forwarding rules29:**end if**

### 5.3. Update Forwarding Rule Based on Predicted Link Status

The description of Algorithm 3 is as follows. In this process, we create new forwarding rules using the predicted adjacency matrix data. By caching forwarding rules that reflect expected link state changes, we maintain low network latency in dynamic networks.
**Algorithm 3** Forwarding Rule Update  1:Update forwarding rules based on predicted adjacency matrix  2:**for** each AP *i* **do**  3:    **for** each AP *j* where j>i **do**  4:        **if** A[i][j]==1 **then**  5:           Establish link connection between AP*_i_* and AP*_j_*  6:        **else**  7:           Calculate shortest path using Dijkstra’s algorithm for rerouting  8:           Establishing forwarding rules for bypass routes  9:        **end if**10:    **end for**11:**end for**12:Remove previous routes and establish new forwarding rules13:**procedure** Dijkstra(graph,source)14:    Initialize distance of all vertices as infinity except the source vertex15:    Set distance of source vertex as 016:    **while** unvisited vertices remain **do**17:        Select the vertex with the minimum distance18:        **for** each neighbor of the selected vertex **do**19:           Calculate tentative distance20:           **if** tentative distance is less than the known distance **then**21:               Update the shortest distance22:               Update the previous vertex23:           **end if**24:        **end for**25:        Mark the selected vertex as visited26:    **end while**27:    Return the shortest path and distance28:**end procedure**

An SDN application performs this task by updating forwarding rules based on the predicted link state. When the link state changes, an event is triggered in the SDN controller, which activates an update process for the communication link state. The SDN controller generates new forwarding rules based on the data from the updated predicted adjacency matrix. The predicted adjacency matrix data are composed of binary values, 0 and 1, which depend on the link state. A predicted link state of 1 means that an active link exists between the nodes. In this case, we create direct forwarding rules for communication between the source (src) node and the destination (dst) node. However, a predicted link state of 0 indicates that there is no direct connection between the nodes. In such cases, an alternative path forwarding rule needs to be created for communication between the src and dst nodes. To achieve this, we use the Dijkstra algorithm to find the shortest path between the src and dst. Then, we create forwarding rules for the lowest-cost alternative path, allowing communication between the src and dst nodes. Afterward, we remove the existing forwarding rules and apply new ones that reflect the predicted link state.

This method pre-caches forwarding rules based on predicted link states, reflecting the relevant information. This allows for quick adaptation to link disconnections in dynamic networks. It helps reduce network latency and improves the stability of data transmission. This ensures reliable communication through low latency, even in environments where the topology changes frequently.

## 6. Validation

In this section, various metrics are used to verify whether the CRIMSON algorithm has reduced latency and request traffic in dynamic networks. In this process, we conduct simulations to compare CRIMSON with other forwarding rule setup methods in dynamic networks. For this, we use metrics such as the confusion matrix, round trip time (RTT) tests, and the count of LLDP packets.

### 6.1. Common Setup

We explain the simulation setup and the details of each process for evaluating the performance of the proposed algorithm, CRIMSON. This evaluation is conducted in a dynamic network featuring mobile SDN nodes. It also includes elements such as communication disconnections and connections that occur in dynamic network as part of the simulation content. We conduct the simulations in various scenarios where the topology changes due to the mobility of nodes with UAV modeling. The details of each setting are shown in Table 1.

#### 6.1.1. Simulation Setup

In this section, we describe the set up of the simulation setting for comparing the performance of CRIMSON. This simulation uses a GCS as the SDN controller and several mobile unmanned nodes. ONOS was used as the SDN controller with version 2.3.0. The mobile unmanned nodes were set up with Mininet-WiFi. We conducted the simulations with five mobile nodes in a $50\times 50\;m^{2}$ area using Mininet-WiFi. We set the prediction interval to 0.01 s to check the real-time network status and topology changes. And we used four bandwidths of 0.5 Mbps, 1 Mbps, 5 Mbps, and 10 Mbps.

#### 6.1.2. UAV Modeling

**Figure 9:**
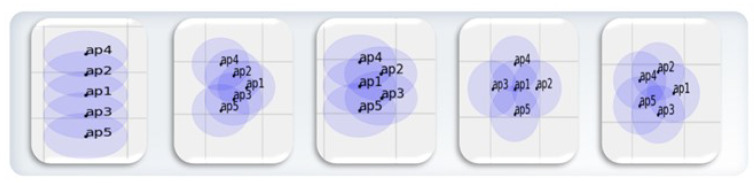
Types of topologies used in the simulation. We use five topologies consisting of five nodes with UAV modeling applied. The topology shapes used, from left to right, are linear, v-shaped, trapezoid, star, and pentagon.

We conducted simulations in a $50\times 50\;m^{2}$ area with mobile nodes using the Mininet-WiFi environment. To enable node mobility, we extracted UAV movement data from rosbag data based on the robot operating system (ROS). Afterward, we converted these data to work with Mininet-WiFi, applying real UAV mobility. We used five nodes with various topologies, including linear, v-shaped, trapezoid, star, and pentagon configurations, as shown in Figure 9. And we set up the environment in Mininet-WiFi to connect or disconnect links based on each node’s coverage area. In this simulation, nodes moved at speeds between 1∼3 m/s based on UAV modeling. Each node’s communication range was set to 5.5 m. Through this setup, we simulated node mobility and communication that could occur in real-world scenarios.

### 6.2. Find the Shiftpoint

In this process, we explain how to find a optimal threshold for detecting changes in cluster movement. The CRIMSON algorithm activates when the changes in the X and Y axes of the cluster exceed the specific threshold. If the threshold is set too high, it does not detect node movement correctly. On the other hand, if the threshold is too low, it may react too sensitively to small movements caused by UAV hovering or wind. Therefore, finding the optimal threshold is important. In this paper, we define this optimal threshold as the Shiftpoint. The goal of this simulation is to use metrics from the confusion matrix, including Precision, Recall, NPV, and Specificity. We aim to find the Shiftpoint by identifying the point where the average of these four values is the highest.

#### 6.2.1. Simulation Setup

**Figure 10:**
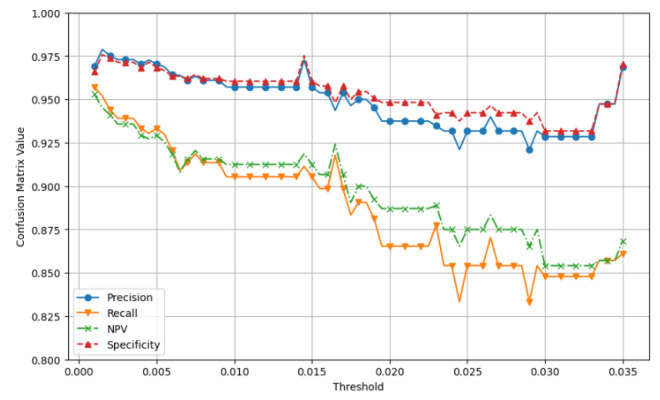
Evaluation of confusion matrix metrics within the threshold range of 0.001 to 0.035. We assess the values of Precision, Recall, NPV, and Specificity throughout this threshold range. Afterward, we select the optimal threshold value that produces the highest average among these four metrics.

The main setup for the simulation is shown in Section 6.1. We tested threshold values from 0.0010 to 0.0350. In Figure 10, we compare the four metrics of the confusion matrix for the predicted link data and the actual data.

#### 6.2.2. Simulation Results

**Figure 11:**
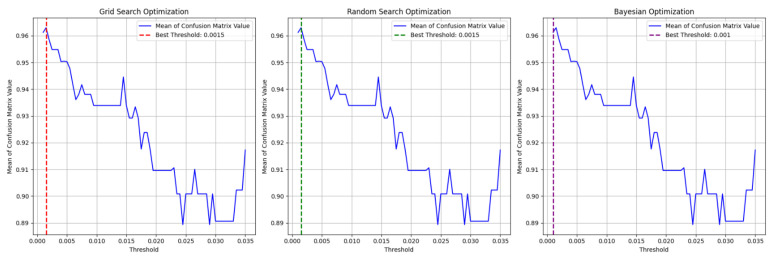
Optimization process for finding the Shiftpoint. This process applies three optimization methods to the average value of the four confusion matrix metrics.

In this simulation, we aimed to find the Shiftpoint by selecting the best option that gave the highest average from the four confusion matrix metrics. For this, we used hyperparameter tuning techniques such as Grid Search Optimization, Random Search Optimization, and Bayesian Optimization. As shown in Figure 11, Grid Search and Random Search found 0.0015 to be the best threshold, and Bayesian Optimization identified 0.001 as the best value. After some evaluation from human and using optimization techniques, we confirmed that 0.0015 is the Shiftpoint.

For the Shiftpoint set at 0.0015, the values from the confusion matrix are Precision 0.979, Recall 0.952, NPV 0.945, and Specificity 0.976, as shown in Table 2. These results indicate that the predicted adjacency matrix accurately indicates the dynamic network.

### 6.3. Network Latency Analysis of CRIMSON

In this process, we compare the dynamic network latency of the proposed algorithm, CRIMSON. The simulation performs the RTT test in a dynamic network where nodes have mobility. The comparison methods are reactive mode and proactive mode.

#### 6.3.1. Simulation Setup

The main setup for the simulation is shown in Section 6.1. We compare CRIMSON’s performance with the reactive mode and proactive mode. For each method, we perform an RTT test in a dynamic network to compare network latency. The reactive mode uses the org.onosproject.fwd app, which is provided by default in ONOS. The proactive mode uses the cobbal.app to establish forwarding rules in advance. For CRIMSON, we created the uds09.app to include the caching of predicted link states.

#### 6.3.2. Simulation Results

**Figure 12:**
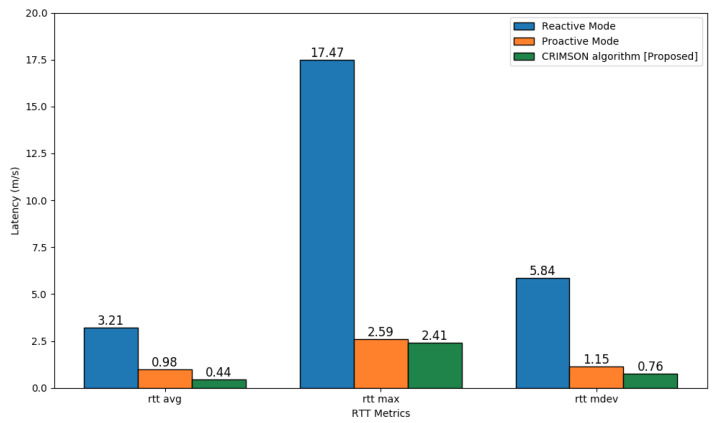
Latency comparison of CRIMSON based on RTT tests. The comparison includes reactive mode and proactive mode. The simulation measured latency using rtt avg, rtt max, and rtt mdev.

The RTT test results, shown in Figure 12, compare the communication latency for different forwarding rule setups. The simulation results indicated that the proposed CRIMSON method reduced average latency compared to the reactive mode and proactive mode, as shown in Table 3. On RTT avg, CRIMSON shows 86.41% and 55.58% lower network latency compared to the reactive mode and proactive mode, respectively. And the maximum RTT demonstrates low network latency by 86.22% and 6.96% compared to the reactive mode and proactive mode, respectively. Additionally, the standard deviation in RTT indicates low network latency by 87.00% and 33.90% compared to the reactive mode and proactive mode, respectively. This indicates that CRIMSON achieves lower network latency than other methods. This means that data reach the destination faster even in dynamic networks.

### 6.4. LLDP Packet Analysis of CRIMSON

In this process, we compare the LLDP transmission count of the proposed algorithm, CRIMSON. LLDP is a protocol that enables the SDN controller and each node to communicate with each other. This allows for the detection of changes in network topology. When the topology changes frequently, the number of LLDP communications for each node increases. This results in an increase in overall network traffic. Therefore, it is important to find a forwarding rule setup that maintains a low LLDP packet count.

#### 6.4.1. Simulation Setup

The main setup for the simulation is shown in Section 6.1. We assess the LLDP count for reactive mode, proactive mode, and CRIMSON. To accomplish this, we measure the number of LLDP packets sent for each forwarding rule system at the SDN controller.

#### 6.4.2. Simulation Results

In this process, we evaluate the number of LLDP packet transmissions in the reactive mode, proactive mode, and CRIMSON. This indicates the average number of LLDP packets transmitted by the SDN controller to each node in a dynamic network.

**Figure 13:**
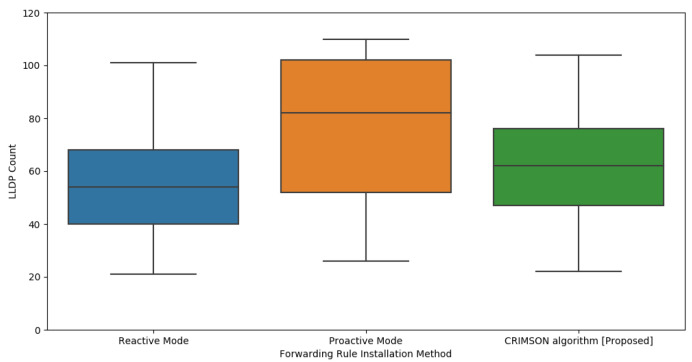
Evaluation of LLDP usage in CRIMSON. The proposed CRIMSON method indicates a lower LLDP count compared to the proactive mode. In an SDN system, LLDP packets are transmitted when packet processing is not handled. This indicates that CRIMSON performs packet processing effectively in dynamic networks.

As shown in Figure 13, CRIMSON has relatively more LLDP transmissions compared to the reactive mode. However, it has fewer LLDP packet transmissions than the proactive mode. This is due to the difference in the method of establishing forwarding rules between reactive and proactive modes. In the reactive mode, forwarding rules are set only when a packet processing request is received. This results in a relatively lower number of LLDP packets compared to the proactive mode. However, in Section 6.3, we confirmed that the reactive mode has higher latency in dynamic networks.

CRIMSON has fewer LLDP transmissions compared to the proactive mode. This is because CRIMSON caches forwarding rules that are expected to change before the topology changes. In an SDN system, if a node does not process a packet, LLDP packets need to be exchanged between the SDN controller and the nodes. Therefore, in the proactive mode, LLDP packets are transmitted whenever network link states change, and nodes do not process packets. This increases the number of LLDP transmissions in dynamic networks where link states frequently change. However, CRIMSON pre-stores forwarding rules for predicted link states. This enables reducing the amount of request traffic.

### 6.5. Comparison of CRIMSON Network Latency at Various Bandwidths

In this process, we compare the network latency of CRIMSON for various types of bandwidth. The quality of the network changes based on the location and distance of the nodes. However, to keep communication stable, it is important to have low latency regardless of changes in bandwidth. In this simulation, we perform RTT tests for each forwarding rule method at four different bandwidth conditions.

#### 6.5.1. Simulation Setup

The main setup for the simulation is shown in Section 6.1. We perform RTT tests in simulated environments with bandwidths of 0.5 Mbps, 1 Mbps, 5 Mbps, and 10 Mbps. We assess reactive mode, proactive mode, and CRIMSON in this simulation.

#### 6.5.2. Simulation Results

**Figure 14:**
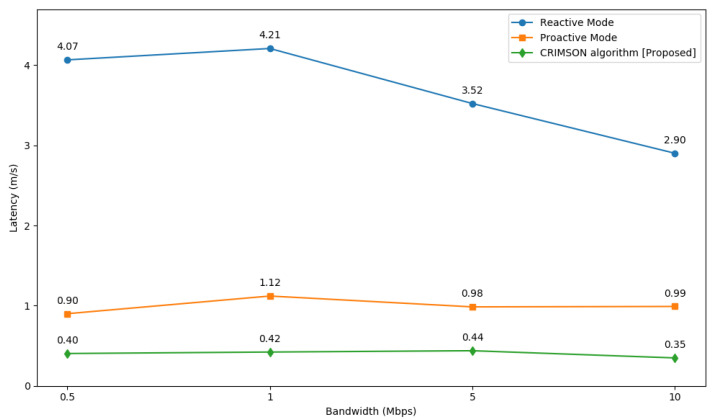
Network latency comparison of CRIMSON at various bandwidths. We conduct RTT tests at 0.5 Mbps, 1 Mbps, 5 Mbps, and 10 Mbps for the proposed CRIMSON algorithm. Simulation results confirm that CRIMSON achieves lower and more stable network latency.

The simulation is executed with various bandwidth conditions of 0.5 Mbps, 1 Mbps, 5 Mbps, and 10 Mbps, as shown in Figure 14. As a result, CRIMSON demonstrates improved performance compared to other methods, as shown in Table 4. CRIMSON has a lower latency than the reactive mode by 90.09% at 0.5 Mbps, 89.98% at 1 Mbps, 87.75% at 5 Mbps, and 88% at 10 Mbps. Compared to the proactive mode, CRIMSON indicates lower latency by 55.21% at 0.5 Mbps, 62.37% at 1 Mbps, 55.54% at 5 Mbps, and 64.84% at 10 Mbps. On average, CRIMSON is about 88.96% and 59.49% better than the reactive mode and proactive mode, respectively. This result indicates that in dynamic networks, CRIMSON still maintains low latency at lower bandwidths compared to other methods.

## 7. Conclusions

This paper proposes the CRIMSON algorithm, which is a caching technique for forwarding rules for predicted link states in SDN. The study aims to reduce latency and request traffic in dynamic networks compared to existing methods. CRIMSON consists of three steps. Through these steps, node mobility and link states are predicted, and then forwarding rules are proactively cached based on these predictions.

The performance evaluation of CRIMSON compares network latency and request traffic through simulations. The comparison is conducted between the reactive mode and proactive mode. In the validation, CRIMSON consistently maintains low latency throughout various bandwidths. The CRIMSON algorithm reduces end-to-end latency by an average of 88.96% and 59.49% compared to conventional reactive and proactive modes, respectively. Furthermore, when comparing the number of LLDP packet transmissions by the SDN controller, the results demonstrate that CRIMSON reduces request traffic more effectively than the proactive mode.

The results of this study indicate that CRIMSON achieves lower network latency and reduced request traffic latency compared to other methods in dynamic networks. This approach has the potential to greatly improve communication reliability and efficiency in future dynamic networks. However, the UAV modeling used in this study primarily assumes movement in a consistent direction when transitioning to different topologies. Therefore, the current CRIMSON algorithm has limitations in environments where node mobility is highly irregular or unpredictable. To address this, future research needs to incorporate machine learning and mathematical modeling for UAV mobility to improve prediction accuracy in irregular movement scenarios. Additionally, it is necessary to explore how CRIMSON performs in various environments by using diverse mobility models and increasing the number of nodes. The goal is to build a reliable dynamic network that improves communication performance and stability in various environments. In future work, we will explore how to adapt the CRIMSON algorithm to environments with varying node densities by incorporating queuing theory to optimize network resource allocation. We will also explore its application to high-bandwidth scenarios, focusing on efficient caching strategies and bandwidth utilization techniques to maintain low latency and reliable communication.

## Figures and Tables

**Table 1:** Simulation settings.

Environment Setting	Detail
SDN controller tool	ONOS
Nodes tool	Mininet-WiFi
Node NUM	5
Mobility model	UAV modeling
Velocity of node	1∼3 m/s
Prediction cycle	0.01 s
Transmission range	5.5 m
Topology shape	linear, v-shaped, star, trapezoid, pentagon
Simulation area	50 × 50 $m^{2}$
Bandwidth	0.5 Mbps, 1 Mbps, 5 Mbps, 10 Mbps

**Table 2:** Confusion matrix indicator value at threshold 0.0015.

Metrics of the Confusion Matrix	Precision	Recall	NPV	Specificity
Value	0.979	0.952	0.945	0.976

**Table 3:** Performance improvement of the CRIMSON compared to other forwarding rule setup methods.

	rtt avg	rtt max	rtt mdev
Improvement over Reactive Mode	86.41%	86.22%	87.00%
Improvement over Proactive Mode	55.58%	6.96%	33.90%

**Table 4:** Performance improvement with bandwidth adjustment of CRIMSON compared to other forwarding rule setup methods.

Bandwidth [Mbps]	Performance Improvement Compared to Reactive Mode	Performance Improvement Compared to Proactive Mode
0.5	90.09%	55.21%
1	89.98%	62.37%
5	87.75%	55.54%
10	88%	64.84%

## Data Availability

Restrictions apply to the datasets.
